# The mechanosensitive Piezo1 channel mechanism of Alzheimer's disease and implications for the development of therapeutic or early detection strategies

**DOI:** 10.3389/fnagi.2025.1707659

**Published:** 2025-12-03

**Authors:** Mo Zhang, Huifang Hou, Hui Li, Xinyang Bai, Huina Song, Lu Wang, Lin-Hua Jiang

**Affiliations:** 1 Department of Physiology and Pathophysiology and Sino-UK Joint Laboratory of Brain Function and Injury of Henan Province, Henan Medical University, Xinxiang, China; 2 Henan Key Laboratory of Neurorestoratology and Protein Modification, The First Affiliated Hospital, Henan Medical University, Xinxiang, China; 3 INSERM U1327 ISCHEMIA ‘Membrane Signalling and Inflammation in Reperfusion Injuries', Faculty of Medicine, University of Tours, Tours, France; 4 School of Biomedical Sciences, Faculty of Biological Sciences, University of Leeds, Leeds, United Kingdom

**Keywords:** Piezo1, Alzheimer's disease, microglia, phagocytosis, synaptic pruning

Alzheimer's disease (AD) is the most common age-related neurodegenerative disease, and the disease mechanism is complex and remains poorly understood. AD is the leading cause of dementia and represents a major clinical challenge. Therefore, early detection and disease-modifying therapeutics are urgently needed. Piezo1 is a mechanically activated Ca^2+^-permeable cation channel with a key role in mechanosensing and mechano-transduction (Xiao, 2024). Recent studies have disclosed a critical role for the Piezo1 channel in modifying AD and, in addition, a positive association of the Piezo1 channel activity in red blood cells (RBCs) with the early development of AD-related dementia. In this opinion piece, we summarize the recent findings and debate whether the Piezo1 channel is a feasible target for formulating strategies for modifying AD and detecting AD-related dementia at the early stage.

## Microglial Piezo1 channel activation by fAβ inhibits AD via stimulating microglial phagocytosis of Aβ

**Figure 1:**
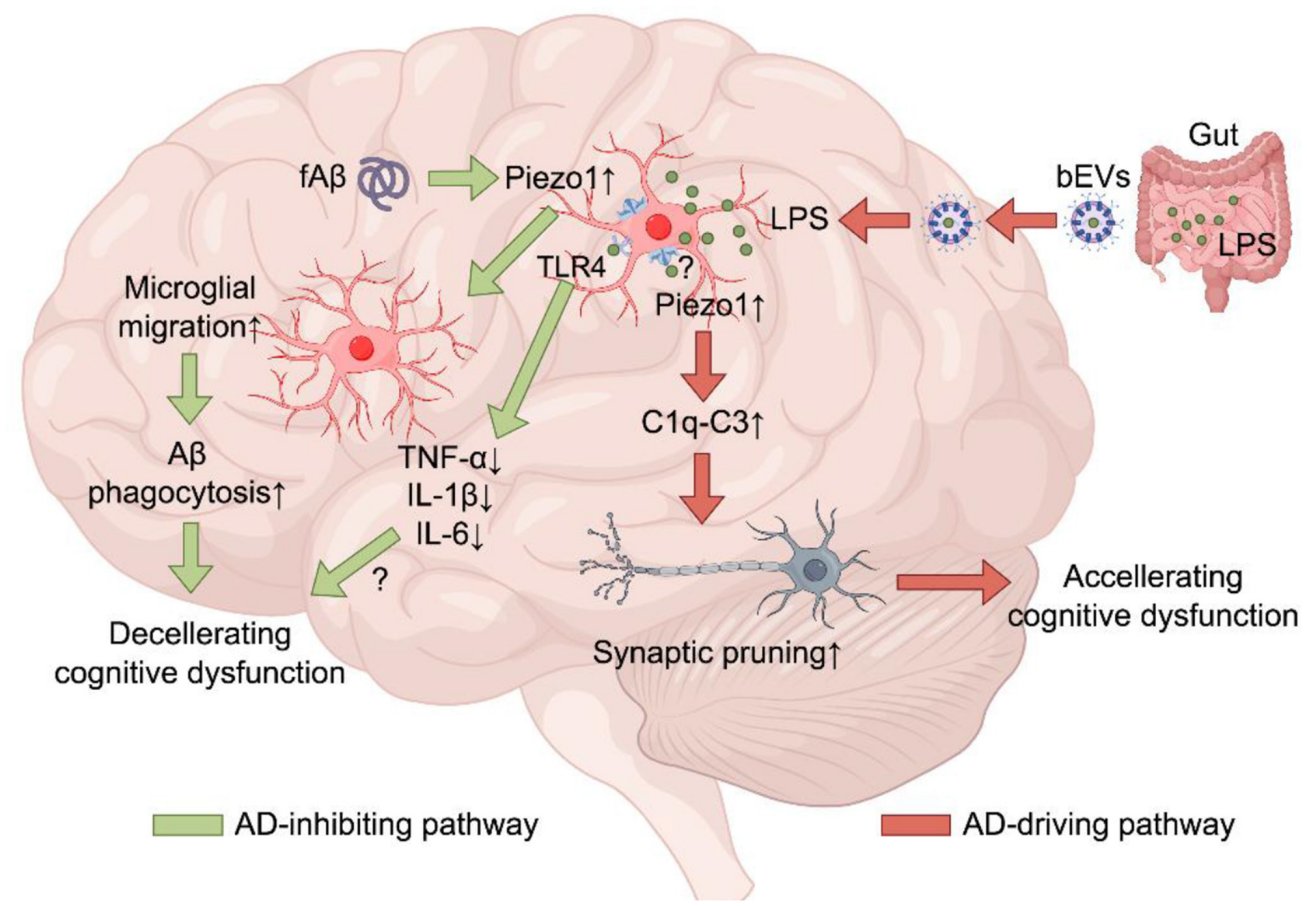
Role and distinctive signaling mechanism of the Piezo1 channel in microglia in modifying AD. Piezo1 channel activation by fAβ promotes microglial migration toward the plaques and enhances the capacity of microglial cells to clear Aβ via phagocytosis, leading to a reduction in Aβ accumulation and synaptic loss, and improvement in cognitive function. In addition, Piezo1 channel activation inhibits LPS-induced TLR4-mediated expression and generation of proinflammatory cytokines, including TNF-α, IL-1β, and IL-6. In contrast, gut bacteria-derived LPS-containing EVs, upon penetrating the gut barrier to enter blood circulation and further crossing the BBB to reach the brain tissue, release LPS to activate the microglial Piezo1 channel via unidentified mechanism(s) to induce the microglial C1q-C3 complemental pathway to mediate synaptic pruning, which has been proposed to contribute to AD-related impairment of cognitive function (the figure was prepared using FigDraw).

Accumulation of amyloid β peptides (Aβ) not only leads to the formation of extracellular senile plaques, one of the pathohistological hallmarks of AD but also acts as an early disease-driving factor. The capacity of microglia to phagocytose Aβ is severely impaired in AD, which plays a critical part in Aβ accumulation and plaque formation. In the plaques, Aβ exists in diverse species, including fibrillar Aβ (fAβ) that significantly stiffens the plaques and surrounding tissues. Interestingly, the Piezo1 channel expression was upregulated in 5xFAD mice, a mouse model of Aβ-driven AD, specifically in a plaque-associated subpopulation of microglia (Jäntti et al., 2022). Consistently, the Piezo1 channel expression was also increased in mouse microglia cultured on fAβ. More importantly, exposure to fAβ induced Ca^2+^ influx via activating the Piezo1 channel (Hu et al., 2023) and, in contrast, the Ca^2+^ response in microglia evoked by the Piezo1 channel activator Yoda1 was inhibited by pre-treatment with soluble Aβ (Jäntti et al., 2022), indicating Aβ species-dependent modulation of the Piezo1 channel. Activation of the Piezo1 channel using Yoda1 stimulated microglial migration and Aβ phagocytosis (Jäntti et al., 2022). As further demonstrated in 5xFAD mice, intraperitoneal injection of Yoda1-enhanced microglial migration and clustering around the plaques and Aβ phagocytosis, alleviated Aβ accumulation and plaque formation and, most importantly, improved cognitive function, whereas deletion of microglial Piezo1 channel expression impaired microglial clustering around the plaques and Aβ internalization, leading to heightened Aβ accumulation and plaque formation and accelerated synaptic and cognitive deficits (Jäntti et al., 2022; Hu et al., 2023). Collectively, these observations provide consistent evidence to indicate that the activation of the microglial Piezo1 channel by fAβ-enriching plaques instigates microglial migration and Aβ phagocytosis, hence limiting Aβ accumulation and plaque formation and improving cognitive function. In other words, the microglial Piezo1 channel plays a vital role in inhibiting AD progression (Figure 1).

## Piezo1 channel activation dampens the proinflammatory response of microglia and astrocytes to Aβ/LPS

In addition to the impaired clearance of Aβ by microglial phagocytosis, chronic and low-grade inflammation, mediated by microglia and also astrocytes, is well recognized as a vital factor in AD progression (Heneka et al., 2025). Emerging evidence points to the glial Piezo1 channels for their role in regulating the proinflammatory response of glial cells to Aβ or lipopolysaccharide (LPS), a neurotoxin released by Gram-negative bacteria. As shown in cultured microglia, exposure to stiff Aβ induced activation of the Piezo1 channel, and thereby promoted microglial generation of reactive oxygen species (ROS) that in turn impaired microglial viability and the capacity of phagocytosis, favoring an important role of microglial Piezo1 channel in the intricate interplays between neuroinflammation and Aβ phagocytosis (Liu et al., 2025). The Piezo1 channel expression was upregulated in TgF344-AD rat brains, mostly noticeable in reactive astrocytes surrounding the plaques in aged TgF344-AD rats infected with *Escherichia coli* (Velasco-Estevez et al., 2020). As shown in cultured astrocytes, the Piezo1 channel expression was upregulated by Aβ-conditioned microglial media, indicating the requirement of microglia in upregulating the astrocytic Piezo1 channel expression (Velasco-Estevez et al., 2020). LPS-evoked proinflammatory response of astrocytes was inhibited by Piezo1 channel activation (Velasco-Estevez et al., 2020). Similarly, LPS-evoked proinflammatory response of microglia, or specifically generation of proinflammatory cytokines, tumor necrosis factor (TNF)-α, interleukin (IL)-1β and IL-6, was inhibited by Piezo1 channel activation (Malko et al., 2023). In microglia, such an inhibition was shown to be mediated by Piezo1-mediated Ca^2+^ influx-dependent suppression of LPS-induced toll-like receptor 4 (TLR4)-mediated activation of the NF-kB signaling pathway (Malko et al., 2023). In short, Piezo1 channel activation dampens the proinflammatory response of microglia (Figure 1) and astrocytes to exposure to Aβ/LPS, but the contribution of these Piezo1-mediated inhibitory mechanisms in AD merits further investigations. Nevertheless, these findings provide more information to endorse and expand the inhibitory role of the Piezo1 channel in AD progression.

## Microglial Piezo1 channel activation by LPS of gut bacterial origin promotes C1q/C3-mediated synaptic pruning and facilitates AD progression

Activation of the microglial complement pathways promotes synaptic pruning, which is a well-recognized mechanism for synaptic loss in the early stage of AD (Tenner and Petrisko, 2025). LPS-containing extracellular vesicles (EVs) released or originated from gut bacteria were detected at a higher level in AD patients than in healthy individuals (Zhao et al., 2025). LPS-containing EVs isolated from human blood (bEVs), upon injection into mice peripherally, penetrated the blood–brain barrier (BBB) and emerged in the brain. Moreover, LPS from such bEVs sufficiently stimulated the microglial C1q-C3 complement pathway to induce synaptic pruning in mice, which was alleviated by depleting microglial Piezo1 channel expression. These observations support the notion that activation of the microglial Piezo1 channel mediates synaptic loss by EV-carried LPS from gut bacteria to the brain (Figure 1). Such a finding sheds novel mechanistic light on the gut–brain axis hypothesis of AD. However, how LPS induces Piezo1 channel activation remains unclear, and evidence for its significance in AD still awaits. It is important to notice that activation of such a microglial Piezo1 channel mechanism facilitates AD progression and thus is opposite to those described above.

## RBC Piezo1 channel activity emerges as a diagnostic indicator for the early detection of AD-related dementia

In addition to the role in modifying AD, emerging evidence suggests an increase in the Piezo1 channel activity in RBCs and supports a positive association of such increased Piezo1 channel activity with the early development of AD-related dementia (Sitnikova et al., 2025). The activity of the Piezo1 channel expressed in RBCs exerts important influences on the volume, shape, and deformability of RBCs, or the ability of RBCs in microcirculation in the brain. Aβ are accumulated in blood vessels and interact with RBCs and, by inducing RBC aggregation, impede microcirculation, leading to cerebral amyloid angiopathy. Pre-incubation of RBCs from healthy donors with Aβ decreased plasma membrane stiffness and viscosity, resulting in higher pressure needed for Piezo1 channel activation (Sitnikova et al., 2025). Acute pre-incubation with Aβ attenuated Piezo1 channel activation, indicating an inhibitory effect on the Piezo1 channel in RBCs (Sitnikova et al., 2025), similar to what was described above for the effect of soluble Aβ on microglia (Jäntti et al., 2022). Exposure to Yoda1 induced similar Ca^2+^ responses in RBCs from both mild cognitive impairment (MCI) and AD patients, which, however, were significantly higher than those in RBCs from healthy subjects. Yoda1-induced cell shrinkage and reduction in the cell size were also greater in RBCs from MCI patients as well as AD patients, as compared to those in healthy RBCs, indicating that upregulation of the Piezo1 expression occurs in MCI or the early stage of AD. Furthermore, interaction analysis, evaluating Piezo1-mediated Ca^2+^ flux in RBCs, conventional AD biomarkers, classification of patients, memory scores, and magnetic resonance imaging (MRI) measurements, revealed positive association of the increase in the Piezo1 channel activity in RBCs with early AD-related dementia, leading to the proposal of incorporating the assessment of the RBC Piezo1 channel activity in diagnostic tests of AD is instructive in the early detection of AD-related dementia (Sitnikova et al., 2025).

In summary, recent studies have disclosed an important role for the mechanosensitive Piezo1 channel in glial cells, particularly microglia, in modifying AD via distinctive mechanisms and, in addition, positive association of an increase in RBC Piezo1 channel activity with early development of AD-related dementia. The findings offer novel mechanistic insights into AD. However, further research is anticipated to gain a better understanding of the Piezo1 channel mechanism of AD and more information to inform whether the Piezo1 channel can be used as a feasible target for formulating strategies to modify AD and detect AD-related dementia at an early stage.
